# Extending bioelectric navigation for displacement and direction detection

**DOI:** 10.1007/s11548-023-02927-w

**Published:** 2023-05-26

**Authors:** Heiko Maier, Heribert Schunkert, Nassir Navab

**Affiliations:** 1 Computer Aided Medical Procedures, Technical University of Munich, Boltzmannstr. 3, 85748 Garching near Munich, Bavaria Germany; 2 Computer Aided Medical Procedures, Johns Hopkins University, 3400 N. Charles Street, Baltimore, MD 21218 USA; 3 Deutsches Herzzentrum München Technical University of Munich, Lazarettstraße 36, 80636 Munich, Bavaria Germany; 4 DZHK (German Centre for Cardiovascular Research), Partner site Munich Heart Alliance, 80636 Munich, Bavaria Germany; 5 Munich Institute of Robotics and Machine Intelligence, Technical University of Munich, Georg-Brauchle-Ring 60-62, 80992 Munich, Bavaria Germany

**Keywords:** Tracking, Navigation, Endovascular interventions, Simulation

## Abstract

**Purpose:**

Bioelectric navigation is a navigation modality for minimally invasive endovascular procedures promising non-fluoroscopic navigation. However, the method offers only limited navigation accuracy between anatomical features and expects the tracked catheter to move only in one direction at all times. We propose to extend bioelectric navigation with additional sensing capabilities, allowing for the estimation of the distance traveled by the catheter, thereby improving accuracy between feature locations and allowing to track also under alternating forward- and backward motion.

**Methods:**

We perform experiments in finite element method (FEM) simulations and in a 3D printed phantom. A solution for estimating the traveled distance using a stationary electrode is proposed, together with an approach on how to evaluate the signals obtained with this additional electrode. We investigate the effects of surrounding tissue conductance on this approach. Finally, the approach is refined in order to mitigate the effects of parallel conductance on the navigation accuracy.

**Results:**

The approach allows to estimate the catheter movement direction and the distance traveled. Simulations show absolute errors below 0.89 mm for non-conducting surrounding tissue, but errors up to 60.27 mm when the tissue is electrically conductive. This effect can be mitigated by a more sophisticated modeling (errors up to 33.96 mm). In experiments in a 3D printed phantom, the mean absolute error over 6 catheter paths is 6.3 mm, with standard deviations smaller than or equal to 1.1 mm.

**Conclusions:**

Extending the setup of bioelectric navigation with an additional stationary electrode allows to estimate the distance traveled by the catheter, as well as the movement direction. The effects of parallel conductive tissue could be partially mitigated in simulations, but further research is needed to investigate these effects in real biological tissue, and to bring the introduced errors down to a clinically acceptable level.

**Supplementary Information:**

The online version contains supplementary material available at 10.1007/s11548-023-02927-w.

## Introduction

The current gold standard for the treatment of many vascular diseases is performing minimally invasive, endovascular surgery. Here, tubular surgical devices such as catheters and guidewire are inserted into the vascular system through a small cut, often in the groin region. Afterward, they are advanced through the vasculature to the region of interest by the surgeon using push and pull motions and rotations. Navigation of these devices is currently performed using a fluoroscopic image stream, constituting a radiation burden to the patient and clinical staff, and forcing the staff to wear cumbersome lead vests during the procedures. Additional injections of potentially nephrotoxic contrast agents are needed to make the vasculature visible.

Among different navigation technologies proposed to overcome the need for fluoroscopic guidance, bioelectric navigation [1] is a novel concept. It employs local, electric fields instead of fluoroscopic images. Despite its potential, bioelectric navigation currently lacks some information that other modalities like fluoroscopy can provide. One example is that it can reasonably detect the catheter position in the proximity of certain vascular features (e.g., bifurcations, stenosis or aneurysms) but lacks localization accuracy between these features. Also, the method as proposed in [1] assumes a monotonous (unidirectional) motion of the catheter, while in real surgeries, catheters are often advanced and withdrawn in alternation.

In this work, we propose to extend the concept of bioelectric navigation with additional sensing capabilities in order to tackle the shortcomings mentioned above. Our approach consists of adding one electrode at a stationary location inside the blood vessel. This could be, for example, at the commonly used vascular access sheath which sits at the location where the vasculature was cut to insert the catheter. We then generate an electric field between this electrode and an electrode on the catheter. Finally, we use the obtained measurements to estimate both the current displacement of the catheter along the centerline of the vascular branch and the current movement direction of the catheter tip. We evaluate this approach in FEM simulations and a 3D printed plastic phantom. In the simulation, we further investigate how tissue surrounding the blood vessel can be detrimental to the performance of the approach and propose modifications to mitigate these shortcomings.

We imagine the proposed distance sensing concept to benefit the adoption of bioelectric navigation into endovascular procedures. There, it could be used as an adjunct to fluoroscopy. Fluoroscopic shots may still be used for tasks such as stent fit validation or checking reperfusion after angioplasty. Meanwhile, the proposed concept could provide non-fluoroscopic catheter localization, thereby reducing radiation dose and contrast agent use.

## Related work

### Bioelectric navigation

Bioelectric navigation [1] is a recently proposed modality for endovascular navigation of catheters. Here, a set of electrodes is placed along the tip of the catheter. A constant amplitude AC current of low frequency (730 Hz) is generated between an electrode at the catheter tip and a ground electrode further down the catheter body. This current generates an electric field which depends on the shape of the blood vessel. Using the same or a different pair of electrodes, the electric field can be probed. Recording the voltage difference between this pair of electrodes over time produces a signal waveform which can be used to detect the current branch of the vasculature the catheter is in. This voltage difference mostly depends on the local diameter of the blood vessel. Thus, when the catheter passes by local geometric vascular features, such as bifurcations, stenosis or aneurysms, or when it moves into a smaller side branch, this produces detectable changes in the waveform. The current vascular branch of the catheter is determined by matching the voltage difference waveform to a set of reference signals simulated from a 3D CT of the anatomy of interest.

So far, the concept has been shown to be able to identify the branch the catheter is currently in, but not the exact location of the tip inside this branch [1]. The approach also assumed that the catheter was monotonously moving forward, while in realistic surgical scenarios, the surgeon might also pull the catheter back and then readvance it (e.g., in order to enter side branches). These are the challenges we aim to tackle within this work.

### Other non-fluoroscopic systems for navigation and tracking

There is a plethora of approaches for non-fluoroscopic tracking of catheters. (For an extensive discussion, see [2].) Conceptually closest to bioelectric navigation are some systems used for cardiac ablation and thus navigation inside the cardiac chambers. Some examples include CARTO 3 [3], EnSite NavX [4] and Kodex EPD [5]. These all differ from bioelectric navigation in different aspects. They either use external field generators for generating a magnetic field, in which the catheter is tracked. Alternatively, electrode patches attached to the patient’s skin (usually 3 pairs which each send electric currents in the x, y or z axis) are used to generate an electric field that is then passively sensed by electrodes on the catheter. The closest to bioelectric navigation is Kodex EPD, in which electric fields are generated between pairs of surface electrodes, but also between catheter electrodes and surface electrodes, meaning that the catheter electrodes do not only act as passive field sensors. Still, Kodex EPD has been only reported for navigation inside the cardiac chambers. In comparison, bioelectric navigation aims at navigating the vasculature (e.g., abdominal aorta or coronary arteries). Also, bioelectric navigation does not use any external surface electrodes, but relies solely on electrodes attached to and moving with the catheter. For our proposed concept, we draw inspiration from the above-mentioned systems using external (stationary) skin electrodes and propose to extend bioelectric navigation by a stationary electrode, placed inside a blood vessel, for estimating catheter displacement and movement direction.

## Methods

### Proposed solution

We aim to tackle the problem that a catheter tracked by bioelectric navigation misses information on movement direction and displacement between anatomical features. We want a solution that we can fit onto a catheter together with the electrodes needed for bioelectric navigation. Then one could detect the current vascular branch, movement direction and exact position along the branch. Thus, we propose an electric sensing concept for direction and displacement, in order to be able to use the same electrodes on the catheter that bioelectric navigation already needs. Ideally, we could use the same electrodes for both tasks to evade adding further hardware complexity to the catheter.

**Fig. 1:**
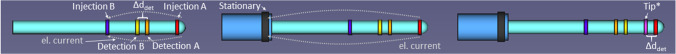
Left: A side view of the tetrapolar configuration for bioelectric navigation. An electric current is generated between injection electrode A (tip electrode, red) and injection electrode B (blue). The voltage difference between the detection electrodes A (orange) and B (yellow) is measured as the bioelectric signal. Middle: For the displacement sensor, we propose to add one stationary electrode (black), e.g., at the vascular access sheath. Right: One additional electrode behind the tip (purple) is used to compensate for parallel conductance and tip movement effects

For bioelectric navigation, we assume a tetrapolar electrode configuration is used. This consists of two current injection and two voltage sensing (detection) electrodes (see Fig. 1, left).

We choose this configuration as it is robust to radial shifts of the catheter inside the vessel (i.e., displacements perpendicular to the centerline) [6]. Here, we propose to extend this setup with an additional electrode that is not attached to the catheter, and is stationary (see Fig. 1, middle). In clinical practice, this electrode could, for example, be attached to the vascular access sheath which stays in place at the site where the surgeon cut the patient’s skin and blood vessel in order to access the vasculature. In this work, we investigate how to estimate catheter displacement (along the centerline) and movement direction using measurements obtained by means of this stationary electrode.

### Local estimation of electric field

Our approach is based on the idea of estimating the electric field locally between the detection electrodes. We take inspiration from the ideas introduced by the LocaLisa system [7] where the authors used six external electrodes attached to the patient skin for tracking the catheter inside the cardiac chamber. We aim to transfer this idea from 3D movement inside the cardiac chamber, using a field generated by electrodes outside of the body, to 1D movement along the centerline of the blood vessel, using electrodes inside the blood stream. In contrast to the LocaLisa approach, in our case one of the field-generating electrodes is moving, i.e., non-stationary, which will have implications discussed later.

We propose to generate an electric current between catheter tip electrode and stationary electrode. This current flow causes continuous voltage drops along its path. Given our two detection electrodes, we can measure the voltage drop between them. We also know the distance between these electrodes $\Delta d_{det}$ by catheter design. We can thus estimate the mean voltage drop per spatial unit between these two electrodes (e.g., 1V per millimeter). If we assume that the catheter body at the detection electrodes is aligned with the centerline of the blood vessel, we thereby obtain an estimate of the mean voltage drop per spatial displacement along the centerline. This is equivalent to estimating the component of the electric field vector along the centerline segment between the detection electrodes:

1 $$\begin{aligned} E_{detA, detB} = \frac{V_{det,A} - V_{det,B}}{\Delta d_{det}} \end{aligned}$$

with $V_{det,A}$ being the voltage between detection electrode A and the stationary electrode, and $V_{det,B}$ being the voltage between detection electrode B and the stationary electrode. This estimated electric field component contains the information of how much the voltage will change if we move a small step along the centerline. If we measure the voltage at detection electrode A at times *t* and $t+\Delta t$, we can then estimate the movement along the centerline within this time frame by

2 $$\begin{aligned} \Delta d_{cath} = \frac{\Delta V_{det,A}}{E_{detA, detB}} = \frac{V_{det,A}(t+\Delta t) - V_{det,A}(t)}{E_{detA, detB}} \end{aligned}$$

Cumulatively summing up these displacements $\Delta d_{cath}$ then gives our estimate of total catheter displacement. Using Eqs. 1 and 2 to obtain these estimates is referred to as approach I from now on.

For now, we implicitly made the assumption that the electric field at the detection electrodes is static, i.e., it does not change while the catheter moves. However, the tip electrode of our catheter, which generates the field, moves together with the rest of the catheter. Our simulations (see ”Simulation results” Section) indicate that as long as the blood vessel is assumed to be surrounded by electrically non-conducting tissue, the assumption of a static field holds true, as all electric current is constrained into the vessel lumen. Under the more realistic assumption that the vessel is surrounded by tissue that can also conduct electric current, though, we observe that the electric field changes while the catheter moves. In this case, the voltage change at detection electrode A is not only influenced by the movement of the electrode inside the electric field. It is also influenced by the movement of the tip electrode which changes the electric field itself, and thus, Eq. 2 does not hold true anymore. We propose to compensate for this by adding one more electrode to the catheter. It is placed slightly behind the tip electrode. We refer to this electrode as $tip^*$ from now on. The distance between tip and tip* is the same as the distance between the detection electrodes, $\Delta d_{det}$ (see Fig. 1, right). We then generate a second electric current between this electrode and the stationary electrode, with a slightly different frequency (e.g., 900 Hz). Given the known distance between the tip and $tip^*$ electrodes, we can now try to estimate the change in voltage at detection electrode A caused by the movement of the tip electrode by a distance $\Delta d_{tip}$:

3 $$\begin{aligned} \frac{\Delta V_{det,A}}{\Delta d_{tip}} = \frac{V_{det,A} - V^*_{det,A}}{\Delta d_{det}} \end{aligned}$$

Here, $V_{det,A}$ is the voltage at detection electrode A generated by the field of the tip electrode, and $V^*_{det,A}$ is the voltage at detection electrode A generated by the field of the $tip^*$ electrode. We assume that the total voltage change at electrode A is the sum of changes caused by tip motion (Eq. 3) and the movement of the detection electrodes (Eq. 2). If we further assume that tip and detection electrodes move by the same distance ($\Delta d_{cath} = \Delta d_{tip}$), we can obtain the compensated formula for estimating catheter displacement as

4 $$\begin{aligned} \Delta d_{cath} = \frac{V_{det,A}(t+\Delta t) - V_{det,A}(t)}{E_{detA, detB} + \frac{\Delta V_{det,A}}{\Delta d_{tip}}} \end{aligned}$$

Using Eq. 4 (together with Eqs. 1 and 3) to obtain displacement estimates is referred to as approach II from now on.

### Simulations

**Fig. 2:**
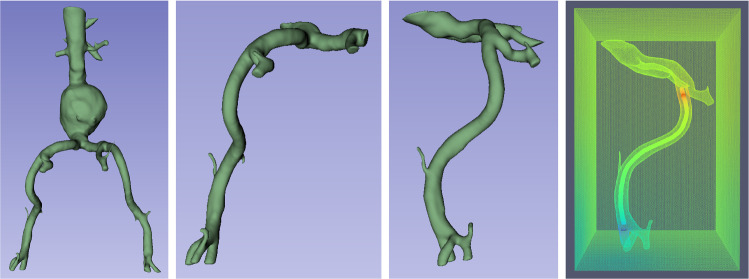
From left to right: Full abdominal vasculature model, cut vascular model in frontal view, cropped vascular model in side view and exemplary FEM solution with the catheter inside the blood vessel, also in side view

To investigate the effects of surrounding tissue on the electric field, we set up an FEM simulation pipeline. As vascular model, we chose the abdominal vasculature of a subject with abdominal aortic aneurysm (AAA), shown in Fig. 2 on the outer left. To speed up the simulations, we cropped one large vessel from the model using 3D Slicer [8], as shown in the inner left and inner right panels of Fig. 2. We also extract the centerline along this vessel using 3D Slicer’s Vascular Modeling Toolkit. We use FreeCAD^1^ to decimate the resulting mesh and convert it to brep format. Gmsh [9] is used for meshing. We model the catheter as a disk extruded along a spline fitted to the centerline. Tissue surrounding the blood vessels is modeled as a cube. The stationary electrode is placed close to the beginning of the vessel (centerline index 10 of 209). For each location on the centerline (starting from index 70), we build a gmsh model by importing the vascular model. Then, we place the catheter with detection electrodes centered around the current centerline location inside the vascular model, and create the surrounding tissue cube. Elmer Solver [10] is used to simulate static current flow using the Static Current Conduction model of Elmer. An exemplary simulation result in one catheter location, color-mapped by the voltage at each point in space, is shown in Fig. 2 on the outer right.

**Fig. 3:**
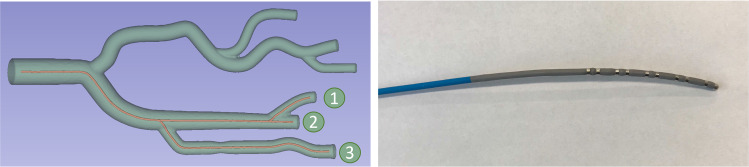
Left: 3D model of the printed phantom, with the paths used for the experiments. Right: Photograph of the catheter used in the experiments

**Fig. 4:**
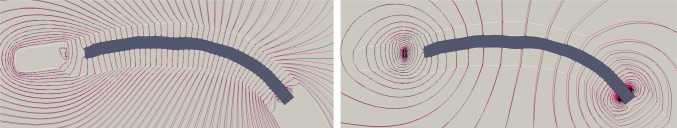
Equipotential voltage lines on a slice through the catheter (dark gray) and blood vessel (white contour), before (purple) and after (black) the catheter moves by 1 mm. Left: In case of surrounding tissue being non-conductive, the electric field inside the blood vessel does not change due to tip motion (purple and black field lines overlap inside the vessel). Right: If the surrounding tissue is conductive, the motion of the tip causes a change in the electric field (purple and black field lines deviate inside the vessel). Notice also that the voltage changes less along the center of the blood vessel, as more current is flowing outside the vessel

### 3D printed phantom experiments

We wanted to validate our displacement estimation in a real setup. A simple vascular phantom (see Fig. 3) was downloaded from a public repository of data associated with [1]. We cut a few branches, rescaled and 3D printed the model. As plastic is not conducting, this phantom corresponds to the case of non-conducting tissue, and we use approach I to estimate displacement. We generated an AC current with a frequency of 1 kHz and a constant amplitude of 100$\mu A$ between tip electrode and a stationary electrode fixed to the vascular tree root. The voltages of detection electrodes and between detection and stationary electrodes were measured using two PicoScope Oscilloscopes (2203 and 3204D). We used a synchronization procedure to synchronize the measurements of the two oscilloscopes and account for slight deviations in their sampling frequencies. This procedure is detailed in the Supplementary Material (Online Resource 1). We used a Webster decapolar catheter (Fig. 3 right) with 5 pairs of electrodes. Each pair is 3mm center-to-center apart (= $\Delta d_{det})$.

**Fig. 5:**
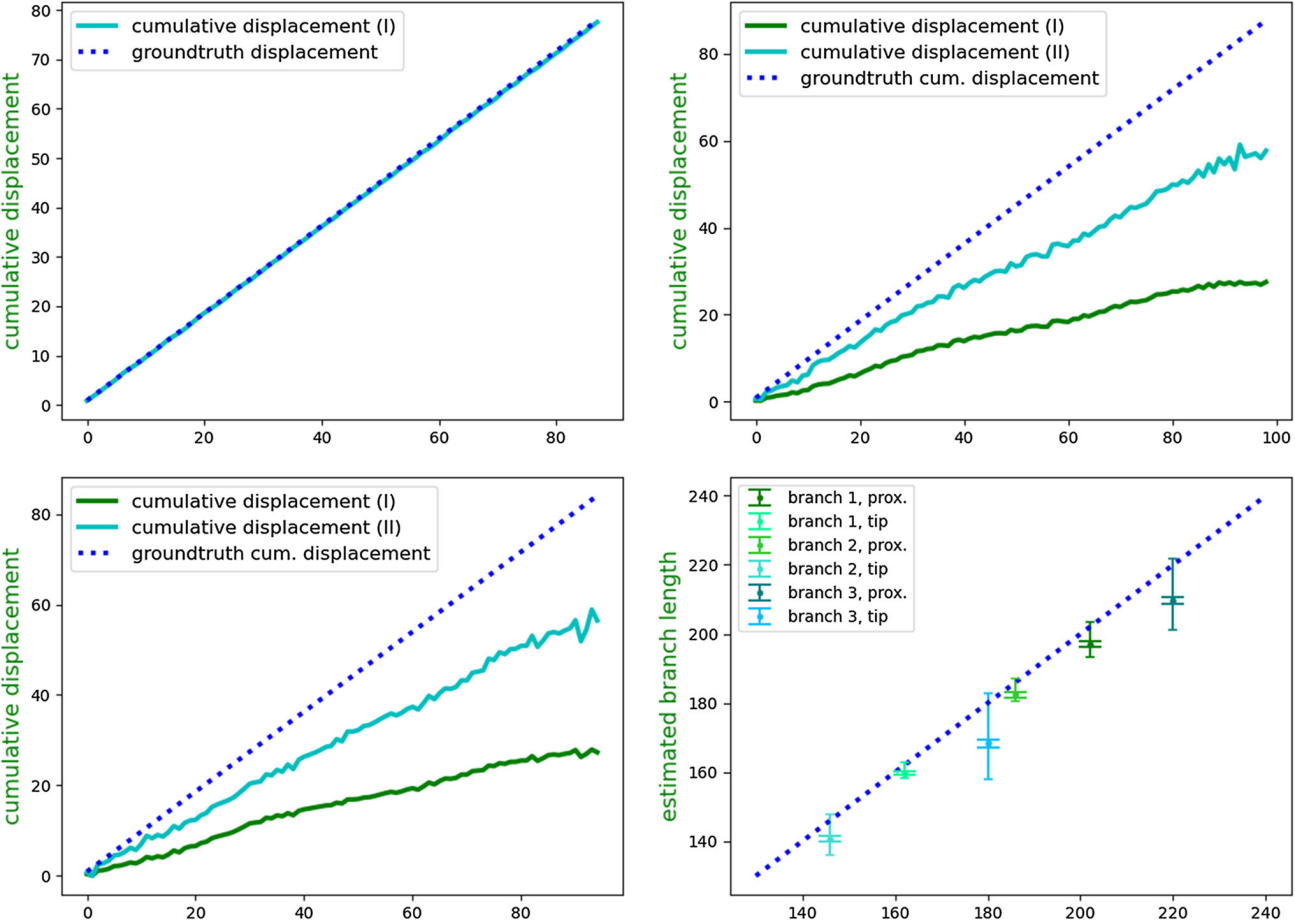
Plots of the simulation results for 100 steps with 0.885mm between subsequent steps (= $\frac{1}{4}$ of $\Delta d_{det}$). Plotted are ground truth cumulative displacements as well as cumulative displacements from approaches I and II. The first three plots from left to right, up to down are corresponding to the first three columns of the first row of Table 1. The lower right plot shows the results of the 3D printed phantom experiments on the 6 paths (cf. Table 2). The inner whiskers represent standard deviation, the outer whiskers minimum and maximum outliers. Notice that the plot is zoomed in (x- and y-axis start at 140mm) for better visibility. For plots 1 to 3, x-axis is simulation step. For plot 4 (lower right), x-axis is ground truth branch length in mm. All y-axes are estimated displacement or branch length in mm

As we know the length of each vascular centerline from the 3D model, we use this as ground truth to validate our displacement estimation approach. For each of the lower 3 branches, we moved the catheter until the most proximal electrode was exactly at the end of the branch. We then pulled back the catheter until the most proximal electrode was exactly at the root of the phantom. The distance traveled by the catheter is thus exactly the length of the centerline of the branch, which we obtained with 3D Slicer. This pullback was recorded 10 times. We then also recorded 10 runs where the catheter was moved until the tip electrode was at the end of the branch, and pulled back until the most proximal electrode was at the root. The distance traveled by the catheter in this case was the length of the centerline minus 4.0 cm (the distance between the centers of tip and proximal electrodes on the catheter).

## Results and discussion

### Simulation results

First, we investigated the effect of surrounding conductive tissue on the electric field during catheter motion. We found that if the surrounding tissue is not conducting, the tip motion of the catheter causes no change in the electric field inside the blood vessel (as assumed in approach I). In case of the surrounding tissue being electrically conductive, the electric field in the vessel changes when the catheter moves (see Fig. 4). This justifies the use of approach II in such scenarios.

Next, we simulated the catheter moving by 100 steps (8.90 cm) along the vessel. We ran simulations for zero tissue conductivity, a conductivity ratio of 0.2 ($=\frac{tissue\;conductivity}{blood\;conductivity}$) and 0.32, the latter one following [11]. We simulated with a distance between tip electrode and first sensing electrode of 2.82 cm and a distance between the sensing electrodes $\Delta d_{det}$ of 0.36 cm and one time with the sensing electrode being closer to the tip (distance of 1.62 cm). We compare the results to ground truth displacements. We tested the performance with the catheter moving at different speeds, with catheter displacement $\Delta d_{cath}$ between subsequent steps varying from 0.885mm (= $\frac{1}{4}$ of $\Delta d_{det}$) to 3.54mm (=$\Delta d_{det}$). The results are depicted in Fig. 5 for $\Delta d_{cath}$ of 0.885mm and Table 1 for all $\Delta d_{cath}$. From the results, it can be seen that approach I estimates catheter displacement accurately when the surrounding tissue is non-conductive, with absolute errors below 0.89 mm for a ground truth displacement of 89 mm (Table 1, column 1). Nonetheless, estimates from approach I strongly deviate from the ground truth displacement if surrounding tissue with nonzero conductivity is present. Using approach II to correct for these effects improves the estimated displacement in all cases with conductive surrounding tissue (Table 1, columns 2 and 3). Approach II still underestimates the displacement, with absolute cumulative errors up to 34 mm. This indicates that effects of surrounding tissue cannot be fully compensated for by this approach, although it mitigates the impact on the estimated displacement. For two different values of surrounding tissue conductivity (column 2 and 3), the estimated displacements are comparable. The results in column 4 further indicate that moving the detection electrodes too close to the tip electrode deteriorates the results. This might be because closer to the tip, the field changes more strongly with tip motion (see Fig. 4). Looking at the errors along the full path of the catheter (Fig. 5), it can be seen that the above-mentioned errors originate from a systematic underestimation of displacements in each time step. These add up along the path, leading to absolute errors that increase with the length of the traveled path.

**Table 1:** Numerical results of simulations. Shown are absolute error of cumulative displacement after 100 steps and RMSE error of cumulative displacement over all steps. Ground truth displacement is 89.0 for all cases. For each pair of rows, the first row shows the results of approach I and the second row of approach II. Rows are grouped by step length as fraction of $\Delta d_{det}$. Column labels are ratio of tissue to blood conductivity $\sigma$ and distance between catheter tip electrode and closest detection electrode $\Delta$ (in cm). All results in mm

		$\sigma$ = 0.0; $\Delta$ = 2.8 cm	$\sigma$ = 0.2; $\Delta$=2.8	$\sigma$ = 0.32; $\Delta$ = 2.8	$\sigma$ = 0.32; $\Delta$=1.6
$\frac{1}{4}$	(I)	0.36 $\mid$ 0.29	60.10 $\mid$ 33.30	56.79 $\mid$ 31.46	75.96 $\mid$ 43.55
	(II)	–	29.93 $\mid$ 15.98	27.61 $\mid$ 14.79	61.72 $\mid$ 34.03
$\frac{1}{2}$	(I)	0.39 $\mid$ 0.31	60.27 $\mid$ 33.34	55.35 $\mid$ 31.39	75.60 $\mid$ 43.47
	(II)	–	33.96 $\mid$ 15.91	23.14 $\mid$ 14.40	62.85 $\mid$ 34.52
$\frac{3}{4}$	(I)	0.89 $\mid$ 0.44	60.20 $\mid$ 34.07	55.40 $\mid$ 31.45	76.12 $\mid$ 44.36
	(II)	–	29.26 $\mid$ 15.81	28.27 $\mid$ 15.18	61.27 $\mid$ 35.31
1	(I)	0.67 $\mid$ 0.40	58.24 $\mid$ 33.26	55.10 $\mid$ 31.25	73.76 $\mid$ 43.22
		–	30.33 $\mid$ 15.58	26.33 $\mid$ 13.50	59.71 $\mid$ 33.79

**Table 2:** Means and standard deviations of estimated path lengths and ground truth path lengths for the phantom experiments. (Prox) stands for starting with the proximal electrode at the branch end, (tip) for tip electrode. Est stands for estimated length, gt for ground truth branch length. Results in cm

	Br1 (prox)	Br1 (tip)	Br2 (prox)	Br2 (tip)	Br3 (prox)	Br3 (tip)
est.	19.7 ± 0.09	16.0 ± 0.06	18.2 ± 0.08	14.1±0.08	21.0 ± 0.10	16.8 ± 0.11
gt.	20.2	16.2	18.6	14.6	22.0	18.0

### Phantom experiment results

The results of the experiments in the 3D printed phantom are given in Table 2 and visualized in the lower right image of Fig. 5. Our displacement estimates show a good overall agreement with the ground truth branch lengths. Nonetheless, it can be seen that they differ from the near-perfect results of the simulation of approach I with non-conducting tissue. While the mean of the 10 runs per setting correlates with the ground truth branch lengths, the estimated length still differs from the ground truth. The mean error varies for different branches between 0.2 and 1.2 cm. The standard deviation of the estimated branch lengths from repeated pullbacks is low, with a maximum of 0.11 cm for Branch 3, indicating good reproducibility. To set these values into clinical perspective, an accuracy of < 5mm is considered sufficient for EVAR interventions, although higher accuracy is needed for fenestrated procedures [12]. Our system cannot meet these accuracy requirements yet.

The results indicate that the approach works not only in simulation, but also in a physical setup. Still, further investigations are needed to determine the source of estimation errors and deviations between repeated runs. There are multiple effects that have not been modeled in the simulations, such as interface effects on the contact surface between electrodes and blood, or the catheter moving off centerline. Potentially one of these effects is the cause for the mismatch between simulated and physical experimentation results.

## Conclusion

Two approaches were proposed on how to utilize a stationary electrode in order to estimate the displacement and movement direction of a bioelectrically navigated catheter. The effect of surrounding tissue on the approaches was investigated in simulations, and finally, one approach was tested in a 3D printed phantom. The phantom tests indicate that the concept does work in a real-world setup, although there remain some errors compared to the simulation results. The absolute error in distance estimation increases with the traveled distance in the presence of conductive surrounding tissue. This suggests that the method might be most suitable for interventions where the distance between static electrode and catheter tip remains rather small. An example could be Transarterial chemoembolisation (TACE) procedures, with the stationary electrode placed on a catheter that rests at a hepatic vascular junction, instead of using a stationary electrode at the access sheath.

One limitation of this work is that the effects of pulsatile blood flow on the impedance of blood [13] have not been modeled, although they might influence the estimated displacements under in vivo conditions.

All proposed approaches underestimate the displacement and thereby drift from the true cumulative displacement over time. In combination with bioelectric navigation, one could compensate for this by readjusting the cumulative displacement whenever the catheter passes a bioelectric feature (e.g., a bifurcation), to prevent the drift from becoming too large. In the future, this work will be integrated with bioelectric navigation and more exhaustively evaluated on phantoms and animal tissue.

## Supplementary Information

Below is the link to the electronic supplementary material.

Supplementary file 1 (pdf 875 KB)

## Data Availability

The 3D printed model is available under https://archive.data.jhu.edu/dataset.xhtml?persistentId=doi:10.7281/T1/4F90KQ. The aorta model is provided by Dr. Petar Valchanov and available under https://sketchfab.com/3d-models/abdominal-aorta-aneurism-4e7aad1805584d9780186017f220fa92 under a CC license (https://creativecommons.org/licenses/by/4.0/).

## Footnotes

1 https://www.freecadweb.org/.
