# Supplementary material for: Extending bioelectric navigation for displacement and direction detection
Source: Int J Comput Assist Radiol Surg. 2023 May 26;18(7):1253–60. doi: 10.1007/s11548-023-02927-w (PMC10329621; doi:10.1007/s11548-023-02927-w)
Supplement: Supplementary file 1 — (pdf 875 KB) [file 11548_2023_2927_MOESM1_ESM.pdf]

Supplementary Material to: Extending Bioelectric Navigation for Displacement Detection

Heiko Maier[1,3,4*], Heribert Schunkert[3,4] and Nassir Navab[1,2,5]

[1*]Computer Aided Medical Procedures, Technical University of Munich, Boltzmannstr. 3, Garching near Munich, 85748, Bavaria, Germany.
[2]Computer Aided Medical Procedures, Johns Hopkins University, 3400 N. Charles Street, Baltimore, 21218, Maryland, USA.
[3]Deutsches Herzzentrum München, Technical University of Munich, Lazarettstraße 36, Munich, 80636, Bavaria, Germany.
[4]DZHK (German Centre for Cardiovascular Research), Partner site Munich Heart Alliance, Munich, 80636, Bavaria, Germany.
[5]Munich Institute of Robotics and Machine Intelligence, Technical University of Munich, Georg-Brauchle-Ring 60-62, Munich, 80992, Bavaria, Germany.

*Corresponding author(s). E-mail(s): heiko.maier@tum.de;
Contributing authors: schunkert@dhm.mhn.de;
nassir.navab@tum.de;

International Journal of Computer Assisted Radiology and Surgery

1 Parameters of Elmer Simulation

- Mesh.MeshSizeFactor = 0.06
- Mesh.MeshSizeMin = 0.0
- Mesh.MeshSizeMax = 20
- Mesh.Format= 2
- Mesh.MshFileVersion = 2.0

- Geometry.OCCFixDegenerated = 1
- Geometry.OCCFixSmallEdges = 1
- Geometry.OCCFixSmallFaces = 1
- Geometry.Tolerance = 0.1
- Geometry.AutoCoherence = 1
- Mesh.Smoothing = 100
- Mesh.MeshSizeFromCurvature = 3
- Mesh.MeshSizeFromPoints = 1
- Mesh.MeshSizeFromParametricPoints = 1
- Mesh.MeshSizeExtendFromBoundary = 1
- Mesh.Algorithm = 6
- Mesh.Algorithm3D = 10
- Mesh.Optimize = 1
- Mesh.OptimizeNetgen = 0
- Mesh.HighOrderOptimize = 0
- Geometry.OCCParallel = 1
- General.NumThreads = 32

2 Synchronization of the Oscilloscopes

The synchronization procedure is designed to account both for a time delay between the measurements of oscilloscope A and oscillscope B, as well as for slight differences in sampling rates. Even when both oscilloscopes are set to the same nominal sampling frequency (e.g. 50 kHz), slight differences in sampling rates can occur when using multiple oscilloscopes. Over time, these can lead to the measurements of the two oscilloscopes slowly drifting out of synchronization.

The basic idea of the procedure is to interrupt the sinusoidal stimulation current at the start and the end of each recording. We do this in order to artificially create features in the recorded waveforms that we can easily identify. We detect these "start" and "end" features in both the waveform recorded by oscilloscope A as well as the waveform recorded by oscilloscope B. We can use the "start" features in order to compensate for a time delay between the start of the recordings of both oscilloscopes. Additionally, we can use "start" and "end" features to identify and compensate for differences in sampling frequencies.

For the latter, we count the number of samples recorded between "start" and "end" by each respective oscilloscope. We expect both oscilloscopes to record the same number of samples in an identical period of time, so any difference in the number of recorded samples must be due to the described difference in sampling periods. From the number of recorded samples, we can thus estimate the difference in sampling frequencies and compensate for it. To create the "start" and "end" features for synchronization, ideally we would have simply switched on/off the stimulation current at two defined points in time. Our oscilloscopes do not support enabling and disabling their output

during a recording, though. Instead, we chose to change the stimulation frequency at two defined points in time, and detect these changes as features for synchronization. More specifically, the procedure works as follows:

1. We start the recording with a stimulation frequency of 800 Hz. After 5 seconds, the stimulation frequency is switched up to 1 kHz ("start" feature), and after 55 seconds, back down to 800 Hz ("end" feature). An exemplary recording containing the switch from 800 Hz to 1kHz can be seen in the Figure below, on the lefthand side. Notice that the switching causes a short interruption in the oscilloscope output. We filter the signals with a 4th order Butterworth bandpass filter, with high and low cutoff frequencies of 50 Hz and 10 kHz, to ease the further signal processing.

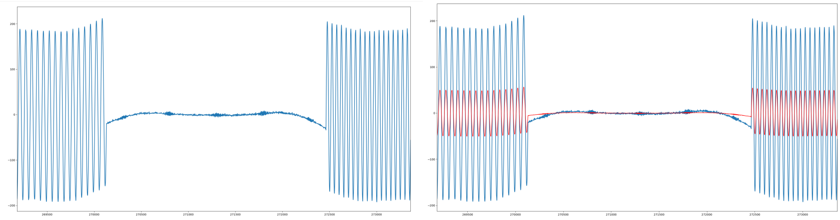

2. We crop two intervals of 0.5 seconds from the signal recorded by oscilloscope A, centered around the points in time where we expect the "start" and "end" features to occur (one at 5 seconds and one at 55 seconds).
3. We find the location of maximum correlation of these intervals with the signal from oscilloscope B. The indices of maximum correlation provide us with two sample indices in signal B that each correspond to one of the indices in signal A from which the intervals where cropped. An exemplary visualization of signals from oscilloscope A and B at their maximum correlation can be seen in the Figure above, on the righthand side.
4. We know the number of samples between the identified indices in the signal from oscilloscope A, N_A. We also know the number of samples between the corresponding indices in the signal of oscilloscope B, N_B. We calculate the sampling period of oscilloscope B, Δt_2, as $\Delta t_2 = \Delta t_1 \cdot \frac{N_A}{N_B}$, with $\Delta t_1 = \frac{1}{50kHz}$. We crop the signals of both oscilloscopes between the "start" and "end" features, to obtain only the segments containing the 1kHz stimulation signal. Then we resample the signal of oscilloscope B with the corrected sampling frequency. Based on these cropped and resampled signals, we then perform the displacement computations as described in the paper.
